# Analysis of Radio Wave Propagation for ISM 2.4 GHz Wireless Sensor Networks in Inhomogeneous Vegetation Environments

**DOI:** 10.3390/s141223650

**Published:** 2014-12-10

**Authors:** Leire Azpilicueta, Peio López-Iturri, Erik Aguirre, Ignacio Mateo, José Javier Astrain, Jesús Villadangos, Francisco Falcone

**Affiliations:** 1 Electrical and Electronic Engineering Department, Universidad Pública de Navarra, Pamplona 31006, Spain; E-Mails: peio.lopez@unavarra.es (P.L.-I.); aguirrerik@gmail.com (E.A.); mateo.58564@e.unavarra.es (I.M.); francisco.falcone@unavarra.es (F.F.); 2 Mathematics and Computer Engineering Department, Universidad Pública de Navarra, Pamplona 31006, Spain; E-Mails: josej.astrain@unavarra.es (J.J.A.); jesusv@unavarra.es (J.V.)

**Keywords:** Wireless Sensor Network, vegetation environment, 3D Ray Launching

## Abstract

The use of wireless networks has experienced exponential growth due to the improvements in terms of battery life and low consumption of the devices. However, it is compulsory to conduct previous radio propagation analysis when deploying a wireless sensor network. These studies are necessary to perform an estimation of the range coverage, in order to optimize the distance between devices in an actual network deployment. In this work, the radio channel characterization for ISM 2.4 GHz Wireless Sensor Networks (WSNs) in an inhomogeneous vegetation environment has been analyzed. This analysis allows designing environment monitoring tools based on ZigBee and WiFi where WSN and smartphones cooperate, providing rich and customized monitoring information to users in a friendly manner. The impact of topology as well as morphology of the environment is assessed by means of an in-house developed 3D Ray Launching code, to emulate the realistic operation in the framework of the scenario. Experimental results gathered from a measurement campaign conducted by deploying a ZigBee Wireless Sensor Network, are analyzed and compared with simulations in this paper. The scenario where this network is intended to operate is a combination of buildings and diverse vegetation species. To gain insight in the effects of radio propagation, a simplified vegetation model has been developed, considering the material parameters and simplified geometry embedded in the simulation scenario. An initial location-based application has been implemented in a real scenario, to test the functionality within a context aware scenario. The use of deterministic tools can aid to know the impact of the topological influence in the deployment of the optimal Wireless Sensor Network in terms of capacity, coverage and energy consumption, making the use of these systems attractive for multiple applications in inhomogeneous vegetation environments.

## 1. Introduction

Wireless sensor networks (WSNs) are emerging as a significant technology with applications in a variety of fields, such as industrial sensing, health monitoring, sports applications, consumer and military applications [1]. With the adequate developments in design and fabrication technologies for ubiquitous wireless connectivity, monitoring of physical and environmental parameters in vegetation environments have been gaining importance in recent years. One of the most important applications of sensing a variety of phenomena such as temperature, relative humidity and smoke in vegetation environments, is for fire detection systems. In [2], the authors show the feasibility of using WSNs for forest fire monitoring. A forest surveillance system for South Korea mountain ranges using sensor networks was designed in [3,4], where the authors present the design and evaluation of a WSN for early detection of forest fires. Another example of forest fire monitoring system based on GPRS and a ZigBee WSN is presented in [5]. WSNs have been also used in a forest environment in [6] for real-time detection of illegal timber logging activities based on sound recognition.

However, the proper design of a WSN for use in large space monitoring could be a very challenging process. When designing any wireless network a relevant aspect to be considered is the maximum distance between two nodes that still ensures a reliable wireless connection. This depends on a variety of parameters such as the transmitter power, the receiver sensitivity, the signal propagation environment, the signal frequency and the parameters of the antennas. Especially, in vegetation environments, the appearance of the foliage in the path of the communication link plays a key role on the quality of service (QoS) for wireless communications systems [7–10]. Discrete scatterers such as randomly distributed leaves, twigs, branches and tree trunks can cause attenuation, scattering, diffraction and absorption of the radiated waves. This severely constrains the design of wireless communication systems in inhomogeneous vegetation environments. The foliage effect on the path loss, shadowing and multipath dispersion has been given considerable attention from the literature. Karaliopoulos *et al.* [7] conducted some empirical foliage loss prediction models for the studies of the isolated foliage effect on a mobile-satellite channel. Bertoni [8] mainly contributed to the studies with the influence of lines of trees along the streets. An excellent work was performed by Rogers *et al.* [9] with a semi-empirical modeling of the foliage loss for the implementation of high speed wireless systems.

The rapid development of WSNs requires a thorough understanding of the wireless communication channels. The functioning of a WSN is clearly dependent upon having adequate signal levels at the distributed nodes, and thus designing effective WSN requires accurate propagation modelling capabilities for complex environments. Because of that, it is highly important to conduct previous radio propagation analysis when deploying a WSN. Traditionally, empirical methods were used (such as COST-231, Walfish-Bertoni, Okumura Hata, *etc.*) [11] for initial coverage estimation. They are time efficient but are less accurate than deterministic methods, which are based on numerical approaches to the resolution of Maxwell's equations. Deterministic methods, such as ray launching and ray tracing (based on geometrical optics) [12] or full-wave simulation techniques (method of moment (MoM), finite difference time domain (FDTD), FITD, *etc.*), are precise but are time-consuming to inherent computational complexity. As a midpoint, deterministic methods based on geometrical optics, offer a reasonable trade-off between precision and required calculation time [13,14].

Deterministic modeling has been used in this work to conduct the characterization of the physical channel for radioplanning purposes in a vegetation environment. The main purpose is to model the radio wave propagation adequately in order to optimize the distance between devices in an actual network deployment, which is based on user location, by means of an in-house generated application which has been tested under real conditions. This paper is divided into the following sections: Section 2 describes the simulation procedure for the channel characterization of the considered scenario. Section 3 shows simulation results such as bi-dimensional planes of received power, power delay profiles and delay spread, among others. Section 4 describes the radioplanning analysis for different technologies (ZigBee and Bluetooth). Section 5 presents the experimental-setup in the vegetation environment and the comparison between simulation and measurements. Section 6 describes the proposed application within the considered scenario, followed by the last section of concluding remarks.

## 2. Ray Launching Technique and Simulation Scenario

A 3D Ray Launching (RL) algorithm has been implemented in-house based on Geometrical Optics (GO) and Geometrical Theory of Diffraction (GTD). Different applications of this algorithm can be found in the literature, like the analysis of wireless propagation in closed environments [15–18], interference analysis [19] or electromagnetic dosimetry evaluation in wireless systems [20].

**Figure 1.**
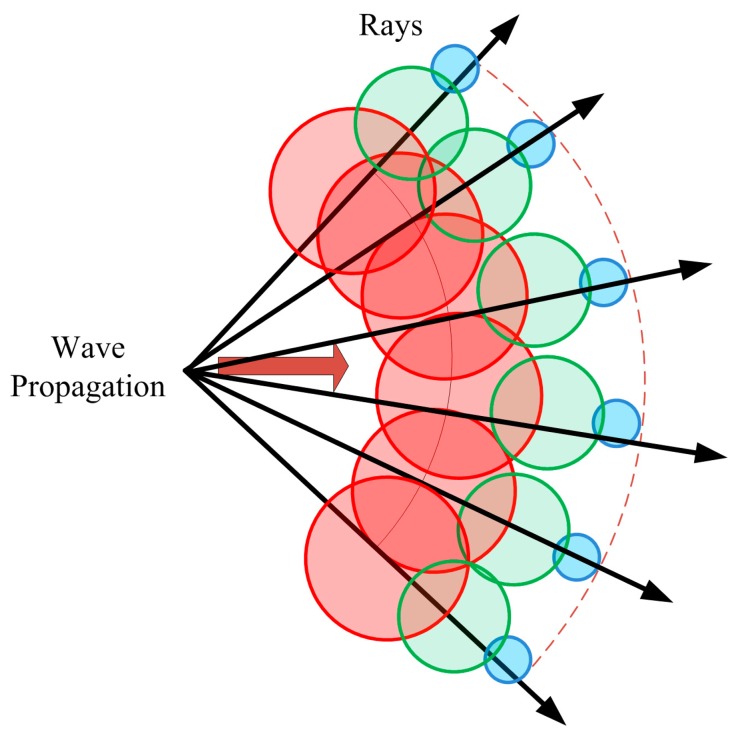
Wavefront propagation with rays associated with single wave front points.

RL techniques are based on identifying a single point on the wave front of the radiated wave with a ray that propagates along the space following a combination of optic and electromagnetic theories, as is illustrated in Figure 1. Each ray propagates in the space as a single optic ray. The electric field *E* created by an antenna with a radiated power *P_rad_* with a directivity *D_t_*(θ*_t_*,Φ*_t_*) and polarization ratio (*X^┴^*, *X^‖^*) at a distance *r* in the free space is calculated by [21]:

(1) $$E_{i}^{⊥}=\sqrt{\frac{P_{\text{rad}}D_{t}\left(\theta_{t},\emptyset_{t}\right)\eta_{0}}{2\Pi }}\frac{e^{-j\beta_{0}r}}{r}{Χ}^{⊥}L^{⊥}$$

(2) $$E_{i}^{∥}=\sqrt{\frac{P_{\text{rad}}D_{t}(\theta_{t},\emptyset_{t})\eta_{0}}{2\Pi }}\frac{e^{-j\beta_{0}r}}{r}{Χ}^{∥}L^{∥}$$

where $\beta_{0}=2\pi f_{c}\sqrt{{ɛ}_{0}\mu_{0}}$, ε_0_ = 8.854 × 10^−12^, µ_0_ = 4π × 10^−7^ and η_0_ = 120π. *L*^┴‖^ are the path loss coefficients for each polarization.

When this ray finds an object in its path, two new rays are created: a reflected ray and a transmitted ray. These rays have new angles provided by Snell's law [22]. The diffracted field is calculated by [23]:

(3) $$E_{\text{UTD}}=e_{0}\frac{e^{-jks_{1}}}{s_{1}}D^{⊥∥}\sqrt{\frac{s_{1}}{s_{2}\left(s_{1}+s_{2}\right)}}e^{-jks_{2}}$$

where *s*_1_, *s*_2_ are the distances from the source to the edge and from the edge to the receiver point, respectively. *D*^┴‖^ are the diffraction coefficients given by [23,24].

**Figure 2.**
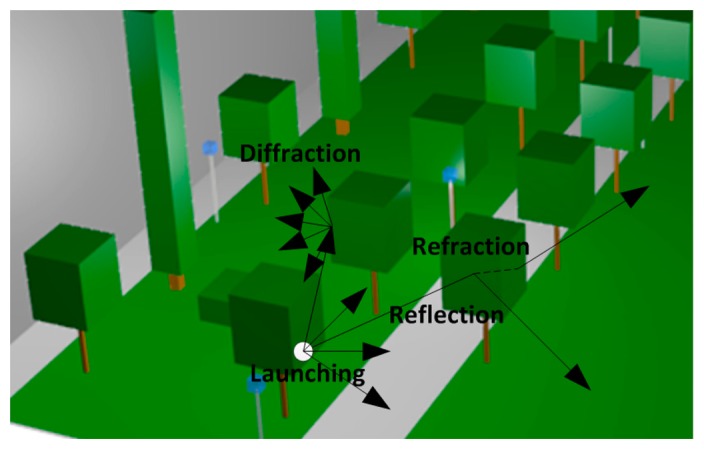
Schematic representation of the principle of operation of the in-house developed 3D Ray Launching algorithm.

The rays considered in GO are only direct, reflected and refracted rays, leading to the existence of abrupt areas, corresponding to the boundaries of the regions where these rays exist. In order to enhance the GO approximation, the diffracted rays are introduced with the GTD and its uniform extension, the Uniform GTD (UTD). The purpose of these rays is to remove the field discontinuities and to introduce proper field corrections, especially in the zero-field regions predicted by GO. Ray launching is performed three-dimensionally, with angular resolution (horizontal and vertical planes) in a predefined solid angle that takes into account the radiation diagram of the transceivers sources. Spatial resolution when computing ray interaction is given by a uniform hexahedral mesh with cuboids of a given lateral dimension. A finite sample of the possible directions of the propagation from the transmitter is chosen and a ray is launched for each such direction. When the ray impacts with an obstacle, reflection, transmission and diffraction will occur, depending on the geometry and the electric properties of the object, as is depicted in Figure 2.

A deterministic method based on an in-house developed 3-D Ray Launching code has been used to analyze the radio electric behavior of an inhomogeneous vegetation environment [25,26]. The software has been implemented in-house based on Matlab programming environment. It is based on geometrical optics (GO) and the uniform geometrical theory of diffraction (UTD), taking into account electromagnetic phenomena like reflection, refraction and diffraction. The main principle of the algorithm is that power is modeled as a finite number of rays launched within a solid angle. Parameters such as frequency of operation, radiation patterns of the antennas, number of multipath reflections, separation angle between rays, and cuboid dimension can be taken into account. Additionally, the material properties for all the elements within the scenario can also be considered, given the dielectric constant and the loss tangent at the frequency range of operation of the system under analysis.

**Figure 3.**
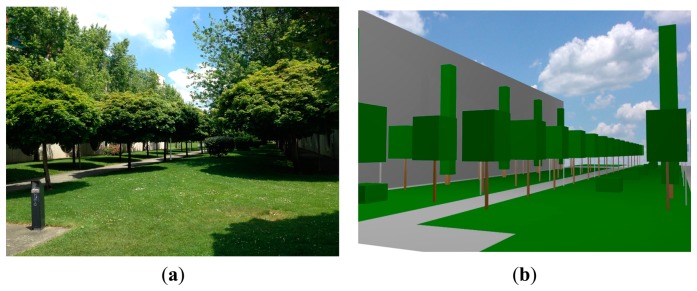
Vegetation scenario under consideration for simulation in the 3D Ray Launching algorithm (**a**) Real view (**b**) Schematic view for simulation.

The simulation scenario implemented for calculation by means of the in-house developed 3D Ray Launching code corresponds to the inhomogeneous vegetation environment represented in Figure 3, which is an outdoor location of the Campus of the Public University of Navarra.

**Figure 4.**
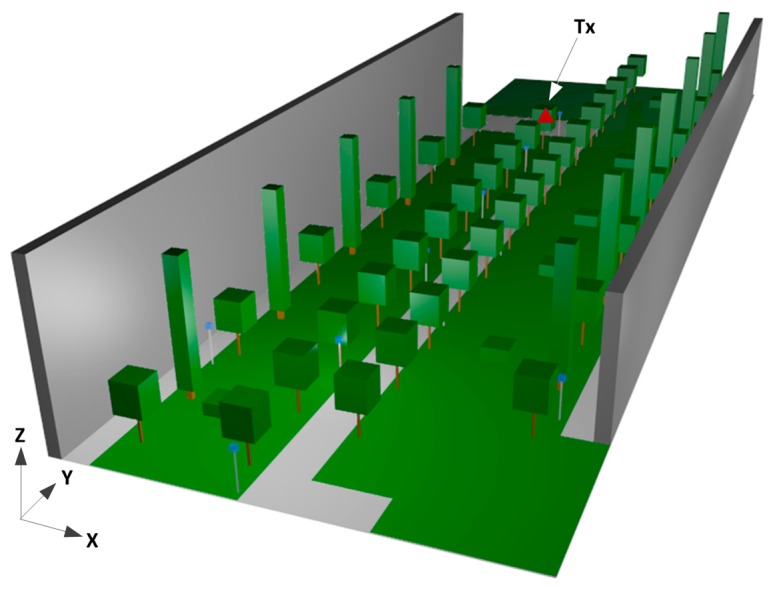
Schematic vegetation environment for simulation in the 3D Ray Launching algorithm.

A view of the complete schematic vegetation model developed for simulation is depicted in Figure 4 with the position of the transmitter. All the material properties for all the elements within the scenario have been considered, given the dielectric constant and the loss tangent at the frequency range of operation of the system under analysis. Due to the continuous changes of the considered environment because of the weather, it is relevant to consider different conditions for the material properties of the trees. The top portion of the tree exhibits high variability depending on the season; for example in winter, there is little foliage whereas in summer the vast totality of the volume of the top of the trees is foliage. In addition, the humidity of the wood of the trunk of the trees strongly varies depending on the weather. This has led us to consider maximum and minimum conditions for the material properties of the foliage and the trunk of the trees. For that purpose, the values obtained in [27] for the material properties of the wood and the foliage have been used. The dielectric constant and conductivity of the wood of the ash tree, which are the trees of the considered scenario is variable with the temperature, as it is shown in Equations (4) and (5) with the parameter *t*, and the dielectric constant and conductivity of the foliage of the trees is variable with humidity, as shown in Equations (6) and (7) with the parameter *h*:

(4) $${ɛ}_{\text{r ash wood}}=-4*10^{-6}t^{3}+0.0002t^{2}-0.0212t+21.483$$

(5) $$\sigma_{\text{ash wood}}=3*10^{-7}t^{3}-0.0003t^{2}-0.004t+7.3238$$

(6) $${ɛ}_{\text{r foliage}}=137h^{3}-69.688h^{2}+23.385h+1.4984$$

(7) $$\sigma_{\text{foliage}}=1.1541h^{3}-0.5489h^{2}+0.1669h-0.0004$$

To set up the dielectric constant and conductivity of the ash wood and the foliage, thresholds for the maximum and minimum humidity and temperature have been considered. These values have been fixed in the interval (0%, 30%) for the humidity and (20°, 40°) for the temperature, according to [27]. Simulations have been performed for the minimum, medium and maximum values and the results shown in this work correspond with the medium values for the humidity and temperature.

The material parameters used in the simulation are defined in Table 1 [28,29].

For the simulations, an antenna has been placed at the point (X = 11.95 m, Y = 70.8, Z = 1.2;, depicted with a red triangle in Figure 4. The radiating element is a wireless ZigBee mote which has been configured as a dipole, transmitting 18 dBm at 2.41 GHz. Simulation parameters are shown in Table 2.

## 3. Simulation Results

**Figure 5.**
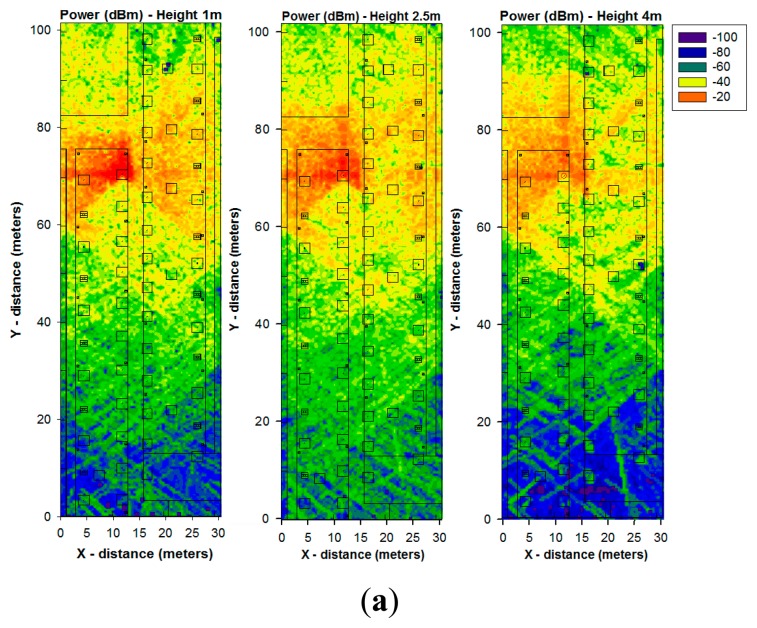

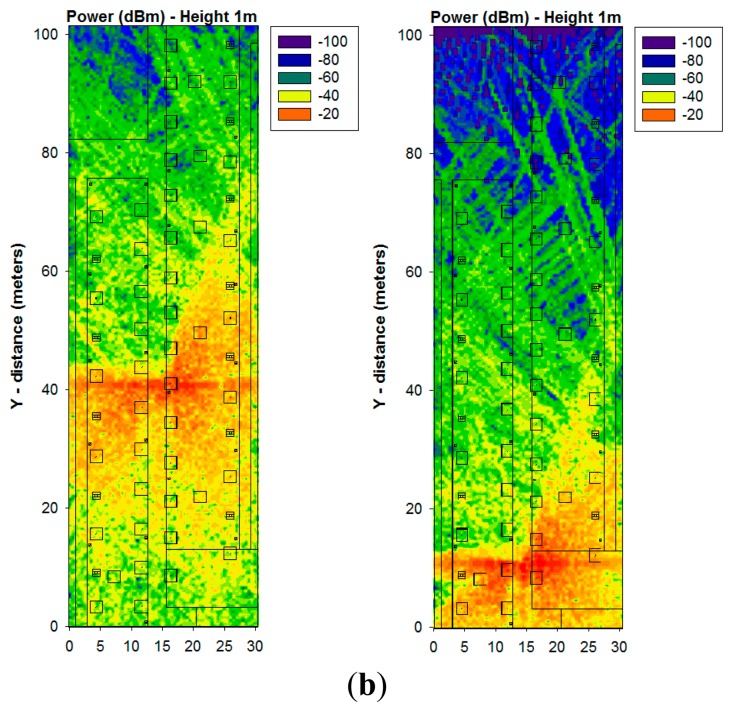
Estimation of received power (dBm) on the vegetation environment obtained by the 3D Ray Launching algorithm (**a**) For the transmitter antenna placed at a fixed point and for different heights (**b**) For two different points of the transmitter antenna for the same height.

Once the simulation scenario has been defined, simulation results can be obtained. Figure 5 shows the power distribution within the considered scenario for different heights (Figure 5a). As it can be seen, the influence of the obstacles (like the trees and streetlights) can be easily appreciated. It is shown that the morphology as well as the topology of the considered scenario has a noticeable impact on radio wave propagation.

In order to gain insight into the influence of the scenario in radio wave propagation, different locations of the transmitter antenna has been considered. Figure 5b represents the received power for 1 m height for two different points (X = 14.7 m, Y = 41, Z = 1.2) and (X = 14.7 m, Y = 11, Z = 1.2;. It is shown that the position of the transmitter antenna plays a key role in the distribution of the received power, because electromagnetic phenomena, such as reflections, diffraction and absorption due to obstacles are different depending of the environment. Therefore, this change in the overall signal levels obtained can provide with mean values of received signal but the consideration of local point values requires analysis for the specific transceivers positions employed.

**Figure 6.**
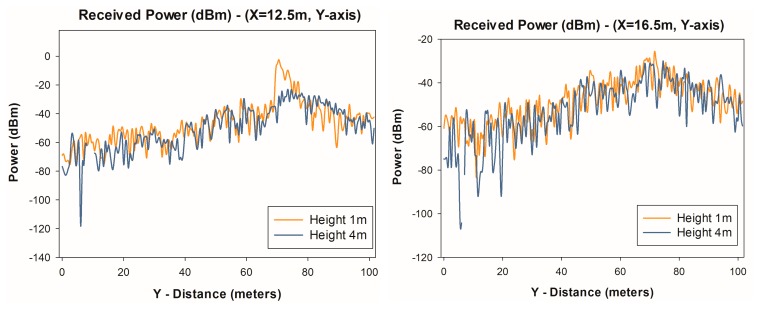
Estimation of radials of received power (dBm) along the Y-axis for X = 12.5m and X = 16.5 m for different heights.

Figure 6 depicts a linear distribution from within the transmitter-receiver range of power along the Y-axis of the considered scenario, for two different values of X, for different heights. It is observed that the distribution of power exhibits large variability due mainly to the strong influence of multipath components.

**Figure 7.**
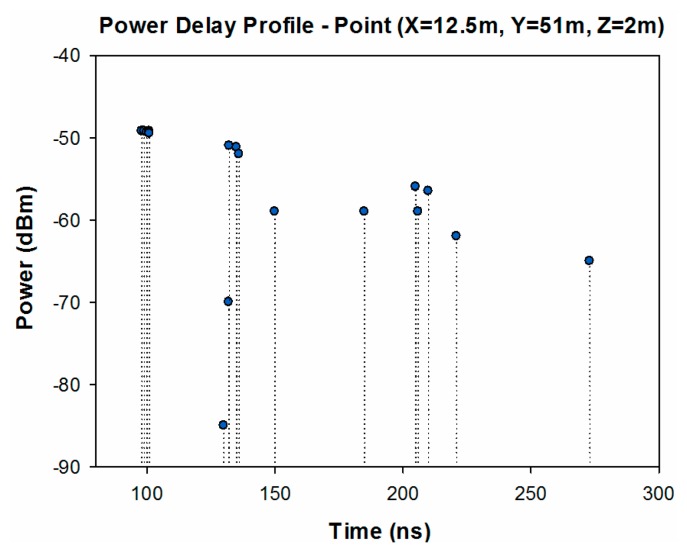
Power Delay Profile at a given cuboid, located at the central location in the considered scenario.

An important radioelectric phenomenon in this type of environment is given by multipath propagation. To illustrate this fact, the power delay profile for the central location of the scenario has been obtained and it is shown in Figure 7. As it is observed, there are several echoes in the scenario inherent to the behavior of multipath channels.

**Figure 8.**
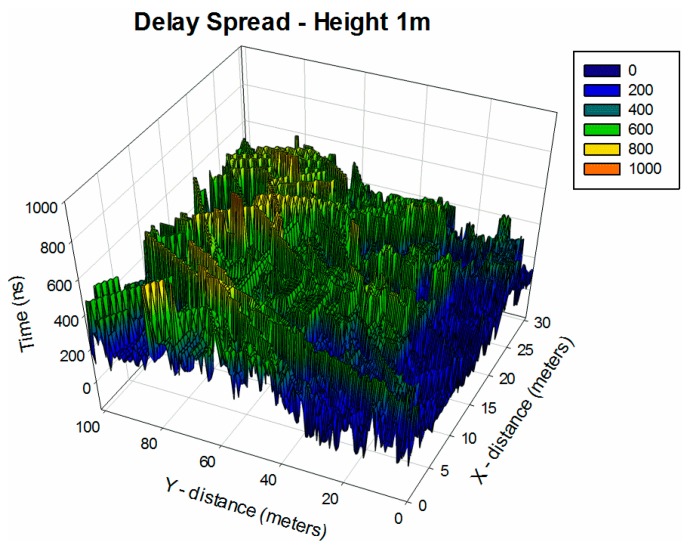
Delay Spread estimation within the considered scenario at 1 m height.

Time dispersion varies widely in a mobile radio channel depending on the geometrical position relationships among the transmitter, the receiver and the surrounding physical environment. Because of that, another parameter that can grossly quantify the multipath channel is the delay spread, which shows the effects of dispersion and is depicted in Figure 8 for the considered scenario.

The strong dependence between the observation point and received multipath components given by the complex morphology of the environment can be clearly seen. Specifically, it is observed that the Delay Spread values nearer the transmitter point are much higher than farther values. This is because at nearer points, the higher power level of the rays produced more dispersion caused by the reflections and diffractions of obstacles and metallic elements inherently presented in the environment.

**Figure 9.**
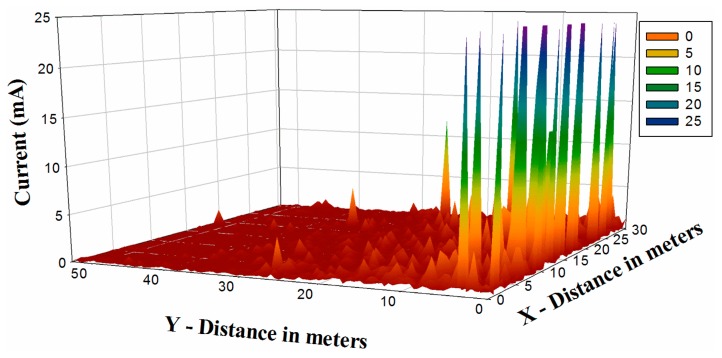
Estimation of energy consumption in terms of current values in mA.

In order to gain insight on the effect of topology on energy consumption, a current consumption map for the considered scenario has been obtained and is shown in Figure 9, for the same position of the transmitter. As it can be seen, the current consumption is strongly dependent on the location of the transceivers, as expected from the received signal variations previously shown.

These results can be used in order to design the optimal network layout, as a function of the number of employed nodes, the variables to transmit (and hence, the required transmission bandwidth) and the resulting sensitivity level. These previous results could be employed in combination with optimization methods in order to estimate the optimal network topology. Moreover, the density of the nodes within the network would have a clear impact on energy consumption, due to the fact that link balance limitations would be lower, since inter-node distance would also be smaller. Care should be taken however, since a larger density of nodes can also lead to increased interference levels, which could degrade system performance. This comment also applies in the case of changes in the transmitted service, associated with a certain allocated bandwidth and hence, changes in sensitivity levels.

**Figure 10.**
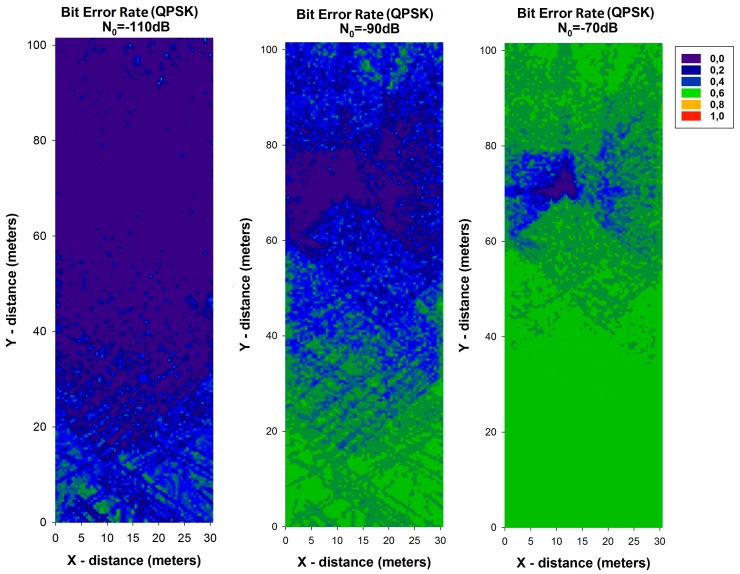
Bit Error Rate for QPSK modulation for different values of N_0_ for data rate of 250 Kbps.

**Figure 11.**
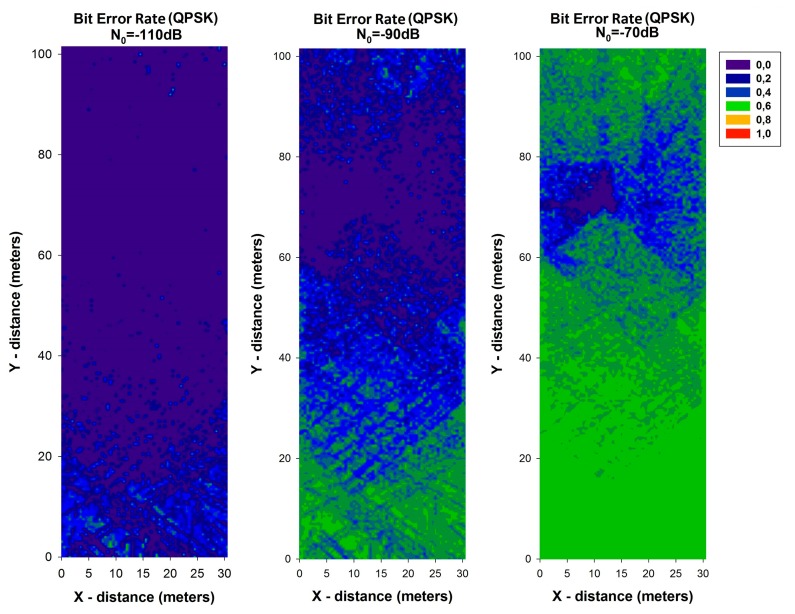
Bit Error Rate for QPSK modulation for different values of N_0_ for data rate of 57,600 bps.

Bit Error Rate (BER) is a key parameter that is used in assessing systems that transmit digital data from one location to another. BER expression for QPSK modulation can be calculated by:

(8) $${\text{BER}}_{\text{QPSK}}=Q\left(\sqrt{2E_{b}/N_{0}}\right)$$

where *E_b_* = *P_RX_*/*R_b_*. The received power (*P_RX_*) has been calculated with the 3D ray launching algorithm for each spatial sample in the considered scenario. With these values, the BER has been calculated and it is shown in Figures 10 and 11, for different values of data rate (*R_b_*) and different values of *N*_0_. It can be seen the high variability between the different cases, with higher values of BER when *N*_0_ is higher. In addition, it is observed the differences between the different data rates considered, leading to a lower BER for all cases with the lowest data rate considered. These results can be very helpful in order to optimize the design and deployment of a WSN depending on the modulation, the used data rate and the level of *N*_0_.

## 4. Radioplanning Analysis

The deployment of two different communication systems has been analyzed within the considered scenario. Firstly, the ZigBee technology has been evaluated. Specifically, the wireless devices used for simulation have been both, the XBee Pro and Xbee motes from Digi International Inc. The differences between them are mainly due to larger transmitter power of the Xbee Pro and its longer range distance. The transmitted power level considered has been reduced to the minimum default value to analyze worst case conditions. Losses due to different weather conditions and foliage have been introduced according to [30], adding 3 dB of losses. In addition, Bluetooth low energy (BLE) and classic Bluetooth have been also evaluated. BLE is an emerging wireless technology developed by the Bluetooth special interest group for short-range communications. In contrast with classic Bluetooth, BLE has been designed as a low-power solution for control and monitoring applications. The transmitted power and sensitivity for the different communication systems considered is shown in Table 3. It must be pointed out that the data of BLE and classic Bluetooth has been obtained from the literature [31].

**Figure 12.**
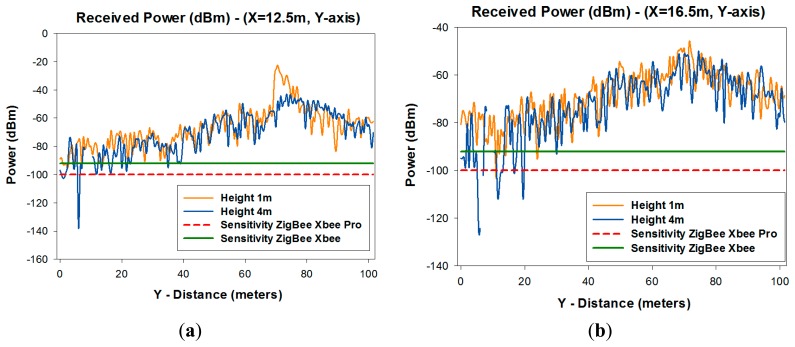

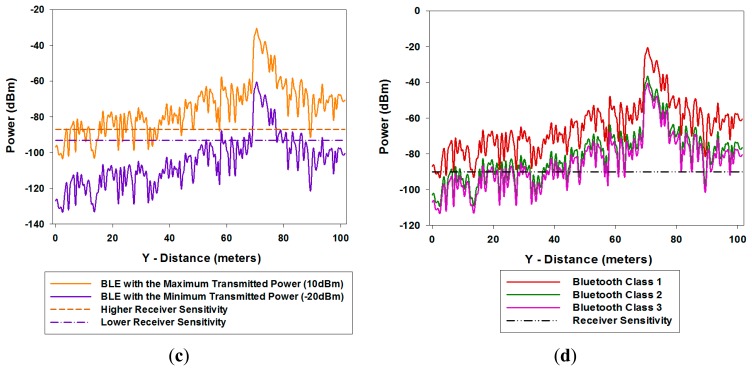
Comparison of radial of received power (dBm) along the Y-axis with the receiver sensitivity (**a**) ZigBee Xbee Pro and ZigBee Xbee for X = 12.5 m (**b**) ZigBee Xbee Pro and ZigBee Xbee for X = 16.5 m (**c**) BLE system (**d**) Classic Bluetooth.

Figure 12 shows the linear distributions of received power along the Y-axis for different values of X for different heights in the case of ZigBee and for one meter height in the case of Bluetooth. Figure 12a,b show the comparison between the received power of XBee Pro and XBee with the sensitivity of each of them.

**Figure 13.**
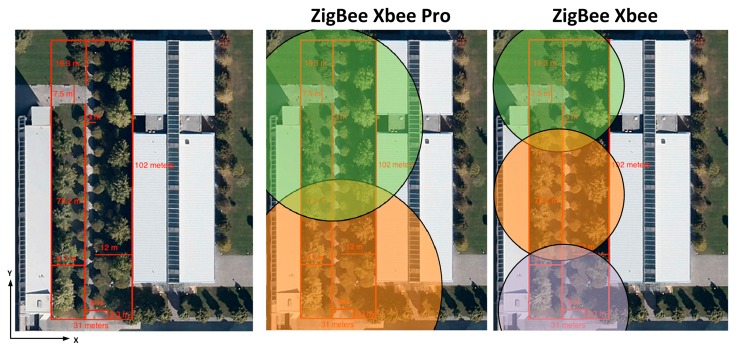

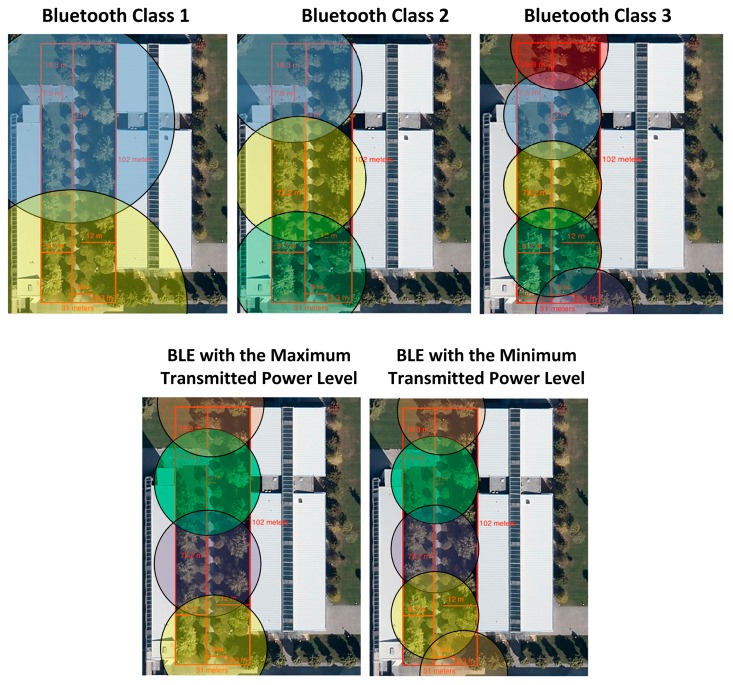
Radioplanning coverage for different technologies within the considered vegetation environment.

Figure 12c shows the comparison between BLE with the maximum and minimum transmitted power and the higher and lower receiver sensitivity depending of which type of BLE has being used. Figure 12d shows the comparison between Class 1, Class 2, and Class 3 of classic Bluetooth. It can be seen that there are some points where the signal goes down below the sensitivity level. With these values, an optimal design of the wireless sensor network coverage can be obtained, as shown in Figure 13.

Another important parameter to be considered when designing a WSN is capacity. Once coverage levels are satisfied, in order to have a good quality of service in the communication link, we must have adequate capacity. The channel capacity is determined by the number of users who are connected to the same communication link at the same time, the data rate of the transceivers and the number of gateways in which the information is gathered.

**Figure 14.**
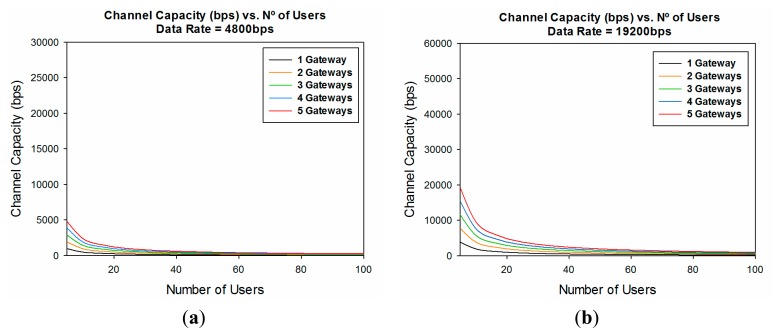

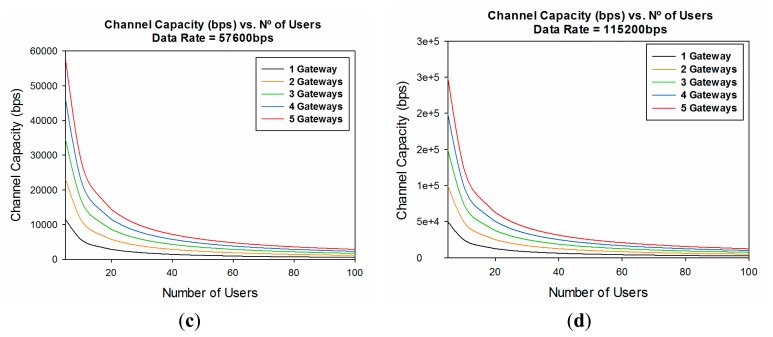
Channel capacity (bps) *vs.* the number of users for different number of gateways considered (**a**) Data Rate = 4800 bps; (**b**) Data Rate = 19,200 bps; (**c**) Data Rate = 57,600 bps; (**d**) Data Rate = 115,200 bps.

**Figure 15.**
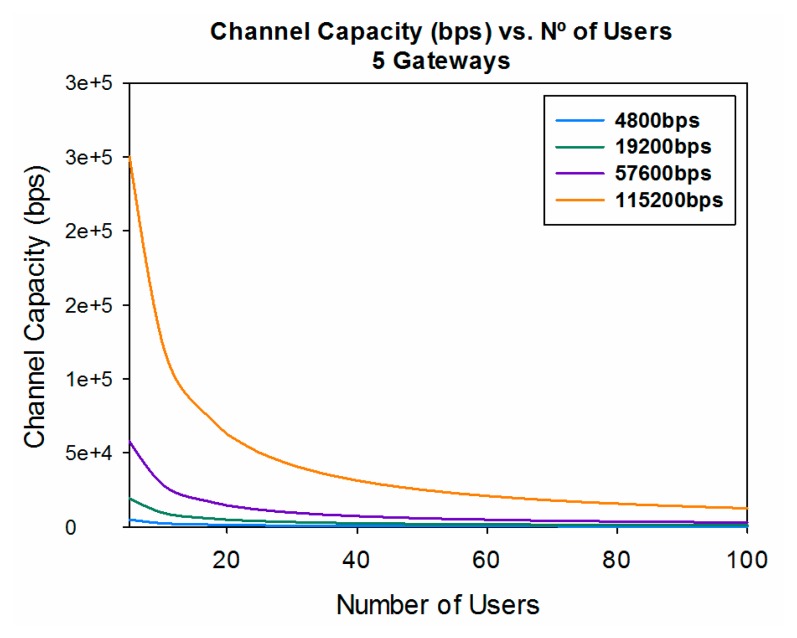
Channel capacity (bps) *vs.* the number of users for different data rates for five gateways considered.

With this information, Figures 14 and 15 show the channel capacity *vs.* the number of users for different data rates considered and for different number of gateways. It is observed as expected that if the information is gathered with a higher number of gateways, the channel capacity increases for the same number of users, for every data rate considered.

It is also very noticeable the great difference in the channel capacity depending of the data rate considered, showing a difference of 29,500 bps between the lowest data rate (4800 bps) and the highest (115,200 bps) (Figure 15). This leads to conclude that, depending of the data rate of the transceivers and the expected number of users it is highly important to adequately fix the number of gateways in the design phase.

## 5. Experimental Setup

**Figure 16.**
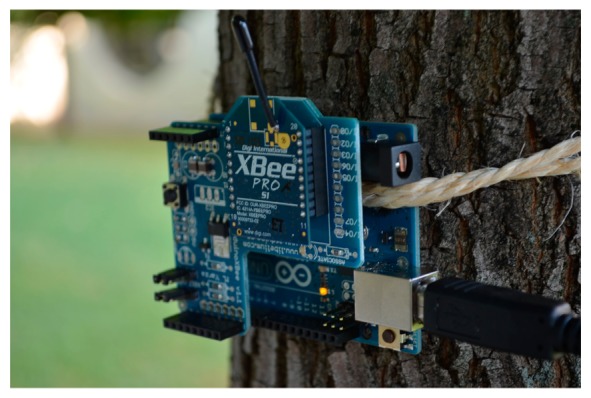
The ZigBee mote used for wireless communications in the vegetation environment.

An experimental setup has been set with the aim of validating the simulation results obtained previously. ZigBee technology has been chosen for emulating a WSN. Specifically, the wireless devices used for the measurements have been the XBee Pro motes from Digi International Inc., shown in Figure 16. These wireless communication devices operate in the unlicensed ISM 2.4 GHz band and the whip antenna mounted on it has an omnidirectional diagram with a gain of 1.5 dBi, which has been taken into account to calibrate the measured values. For transmitting or processing received data, the motes have been connected to a PC via USB cable after being plugged into an XBee explorer unit.

Two different measurement campaigns have been carried out. In first place, the radio propagation in the vegetation environment has been characterized setting a transmitting ZigBee mote on the trunk of one of the trees, at the point indicated in Figure 4. The points of measurement are the rows of trees along the path, with the receiver placed inside the foliage of each tree. The received power level has been measured by means of an Agilent N9912 Field Fox portable spectrum analyzer. The omnidirectional antenna coupled to the analyzer operates at ISM 2.4 GHz band and has a gain of 5 dBi. The parameters of the transmitting ZigBee mote can be seen in Table 4.

**Figure 17.**
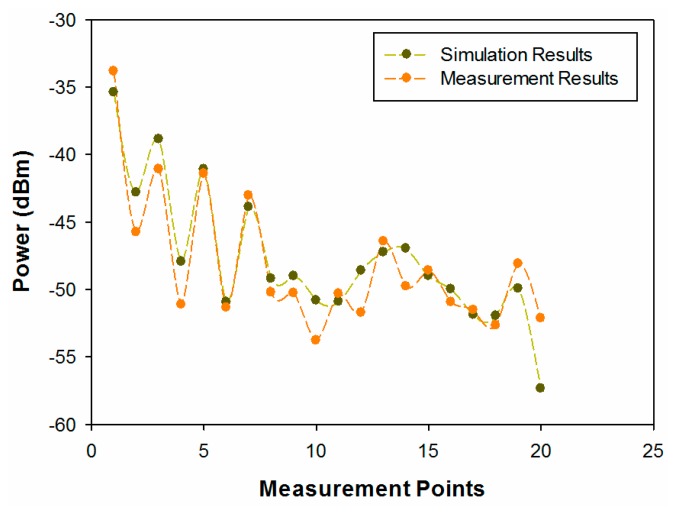
Comparison of simulation *vs.* measurements.

The measurement results for received power in the locations previously described can be seen in Figure 17, where 3D ray launching simulation results have also been included for comparison.

Good agreement is observed between the simulation results and the measurements. The resulting error mean for those measurement points is 1.67 dB, a low error that indicates that the proposed in-house 3D ray launching simulation method works properly, validating in the same way the simulation results shown in the previous sections of this work. Once the received power level for different positions within the vegetation environment has been measured and the simulation method has been validated, the second measurement campaign has been performed. The aim of these measurements is to deepen the analysis of the radio propagation in a highly complex environment such as the considered scenario. For that purpose, the quality of the ZigBee channel has been measured. Specifically, the value of Packet Error Rate (PER) has been used as quality parameter, which indicates the percentage of transmitted packets that has been lost and do not reach properly the receiver. The position of the transmitter and the measurement points have been the same as in the previous measurement campaign. But in this case, another XBee Pro mote has been placed on the measurement points in order to implement ZigBee radio links. In addition, in this case, two different measurements have been done for each tree, one with the XBee Pro mote placed at the trunk of the tree and the second one with the mote placed in the foliage of the tree. Table 5 shows the configuration of the wireless mote's parameters used for measuring the PER. Notice that ACK options have been disabled to avoid packet retransmissions and the transmitted power level has been reduced to the minimum default value in order to analyze the worst case, in which the lost packet quantity will be the highest. The PER value has been calculated transmitting 100,000 packets. Two in-house developed applications based on Java, one for the transmitter and the other for the receiver, have been used in order to configure easily the parameters shown in Table 4, as well as to calculate the PER by reading the sequence number of the received packets.

**Figure 18.**
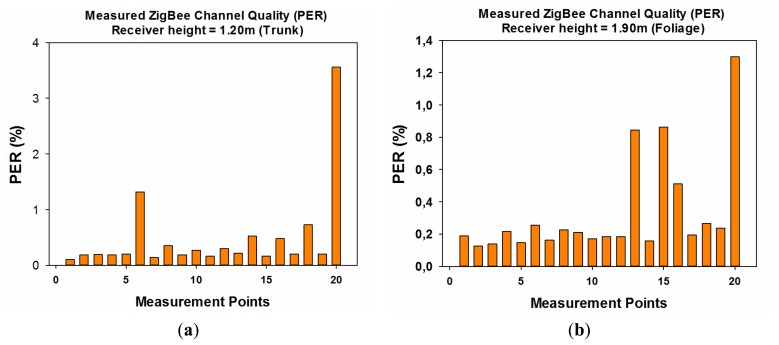
ZigBee wireless channel quality measurements in the considered scenario (**a**) Receiver placed at the trunk of the tree (**b**) Receiver placed within the foliage of the tree.

The measured values of PER are shown in Figure 18. As can be seen, the values are, for every case, lower than 5%, so the number of lost packets is very low. It is worth noting that PER depends strongly (but not exclusively) on the received power level. Taking into account that the measured PER results have been obtained with the transmitted power level set to the lowest value, ACK option disabled and a quite high transmission rate (as for a real ZigBee applications lower rates are usually needed), it can be concluded that no channel quality problems in terms of packet losses would be within this kind of scenarios. This is consistent with previous power level estimations obtained in simulation as well as by direct measurement, where in all cases the values are above the threshold values for conventional QPSK modulation schemes. In this way, probability of error is low, leading to a small amount of lost packets. Furthermore, PER could be improved increasing transmitted power level, activating ACK options or reducing transmission rate.

## 6. Application

**Figure 19.**
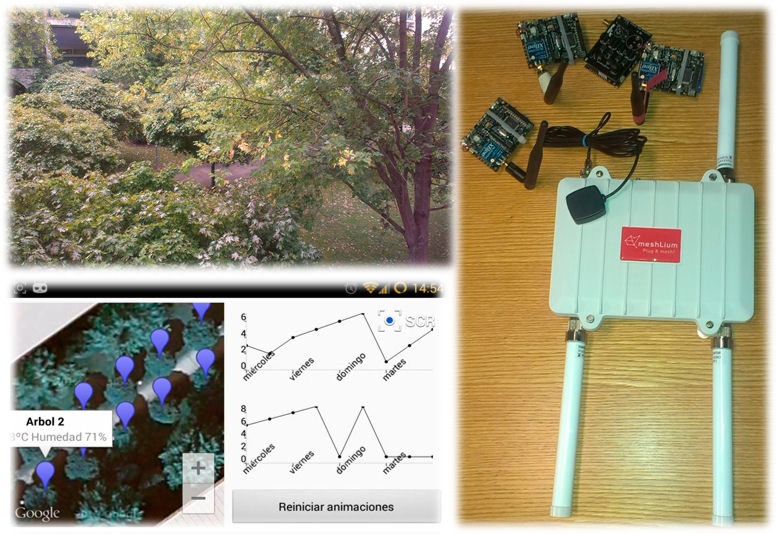
Experimental scenario (**left top**), application (**bottom left**) and devices of the WSN (**right**).

Once a complete model for the characterization of radiowave propagation has been developed, a real test scenario in terms of end users and potential application layer is the next goal to provide a holistic view. With this aim in mind, an Android-based application has been implemented, devoted to monitor certain environmental conditions of the campus, although the system is scalable in order to include more variables, such as social based applications or perimetral security. The system consists on a WSN of ZigBee motes and a Meshlium device. This device is a Linux based micro-PC with an ×86,500 MHz processor and 256 MB RAM memory. It also includes four wireless communication interfaces, including in our case WiFi (2.4 and 5 GHz), ZigBee and GPS. This device has been designed to operate outdoor and can be easily emplaced in many locations since its power supply may also be provided by a solar panel and a battery. Figure 19 illustrates the scenario (left top), some components of the WSN (right side) and the application interface (bottom left).

Motes, in charge of ambient intelligence collection, are embedded on the trees ride of the campus and also on lampposts. They gather environmental information (temperature, atmospheric pressure, contamination levels −CO, CO_2_, NO_2_, O_3_−…) and also noise information in order to build noise maps. The information is collected periodically, following a programmable frequency, and transferred to the Meshlium, which includes a MySQL database and a web server in charge of data storing and information publishing respectively.

The service oriented application developed has two main services: a PUSH alert service, and an on-demand (PULL) monitoring service. The alert service allows the definition of certain parameters to be continuously monitored so that when thresholds previously defined are reached, the system notifies this contingency to the user interested in that alert. Each of the users subscribed to this service can define as many alert profiles as deemed appropriate. If an alert is triggered, the user is immediately alerted, thanks to the PUSH conception of the service, on his/her mobile device (smartphone or tablet). The monitoring service allows users to observe environmental conditions in real time and also to analyze previously happened events. Users have access to noise and contamination maps, statistics, graphical charts, periodical reports and twilight data. In its current version, the application is only available in Spanish but, thanks to its multilingual design, it is easily adaptable to other languages.

In order to grant scalability, data aggregation is performed in two stages. A local stage, which is performed at each mote, concerns data gathering from all the sensors of the mote following a predetermined sequential order fixed in advance to minimize the number of sensing operations. Once each mote has collected its information, the second stage is initiated. The second stage concerns data aggregation from all the motes of the WSN, something done by means of a chain aggregation algorithm which follows a space filling curve and ensures the exchanging of a minimum number of messages. The aggregation chain is initially built at the deployment stage and remains unchanged until the one of the nodes present some kind of persistent malfunction or run out of battery. Then, a chain reconstruction algorithm is triggered, and normal operation of the network is restored. Reconstruction is a somewhat complex process that is rarely triggered. Normal operation implies that each mote only receives a message from its predecessor in the chain and only sends a message to its successor.

Communication among sensors and between sensors and Meshlium is performed using ZigBee interfaces, while communication among Meshlium and smartphone and tablets is performed using WiFi technology.

**Figure 20.**
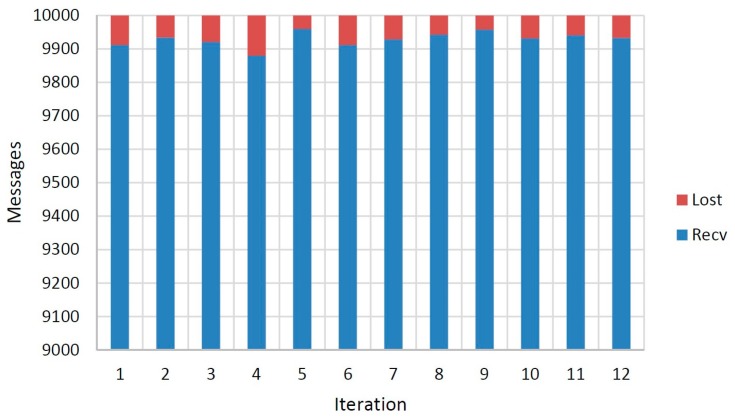
Messages received and lost during the experimentation.

The experimental scenario has been covered with a chain of eight nodes, one of them acting as chain initiator (node 1) and another one acting as gateway. In order to evaluate different environmental situations, a dozen of experiments have been conducted on different dates and environmental conditions, obtaining minimal differences between them. Each experiment implies that the initiator node sends a message to its successor every 10 s, up to a total of 10,000 messages. During each cycle, messages travel from a given mote, where information is aggregated, to its next one until the sink (gateway), which transmits the message to the Meshlium device where information is stored. The loss of a message along the chain implies that the cycle is not completed successfully. Figure 20 illustrates the results obtained during the experimentation. It is observed that only 845 of the cycles (0.70%) are not accomplished successfully. According to the dozen of experiments performed, one can observe an average of 0.70% of messages lost over the total of messages sent, with a standard deviation of 0.22% and a median of 0.68%, implying a very low level of loss. Network has been constructed so that if the gateway node detects that after ten consecutive cycles without accomplishing any iteration, the gateway node triggers an automatic process in charge of chain reconstruction. However, this loss condition has not occurred during the experimentation, so the chain reconstruction process has never been triggered.

**Figure 21.**
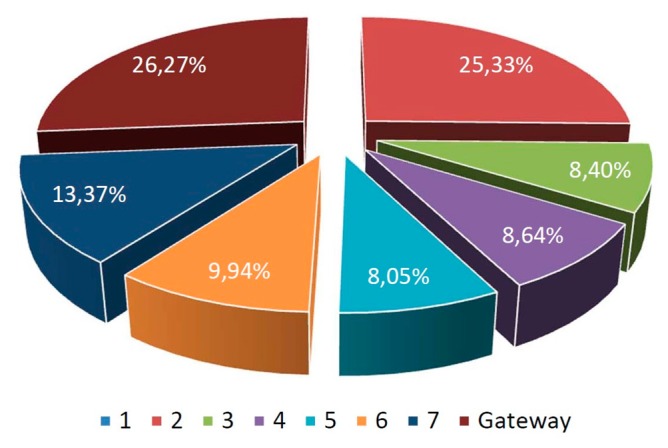
Rate of messages lost by node.

Figure 21 shows the rate of messages lost by node. Note that the first mote never loses any message since it acts as initiator of the chain.

Although loss rate is very low, one can note that two motes have a failure rate that is double than others. The second mote and the last one act as stoppers, the first one due to the initial flow of the network, and the last one due to its status of gateway to the Meshlium. In both cases, one can observe a limited contention effect, since motes do not have multithreading capability, and therefore, they must serialize their tasks.

## 7. Conclusions

In this work, the demands for modeling the radio channel in inhomogeneous vegetation environments are presented. The obtained results show the complexity of vegetation scenarios. It can be concluded that the morphology as well as the topology of the scenario plays a key role in the estimation of radio signal propagation, due to the strong impact of multipath components in the overall loss mechanism of the propagating radiowave. The agreement between simulation and measurement results validates the 3D ray launching algorithm, making it adequate for radioplanning analysis with the aim of deploying WSN within this type of environments. Moreover, a detailed characterization and analysis of the radio wave propagation for ISM 2.4 GHz wireless sensor networks in heterogeneous vegetation scenarios allow to design environment monitoring tools based on ZigBee and WiFi technologies where WSN and smarthpones cooperate providing rich and customized monitoring information to users in a friendly manner, enabling context aware scenario implementation.

## Figures and Tables

**Table 1.** Material properties in the ray launching simulation.

**Parameter**	**Permittivity (ε_r_)**	**Conductivity (σ) [S/m]**
Air	1	0
Concrete	5.66	0.142
Grass	30	0.01
Trunk tree	Equation (4)	Equation (5)
Tree foliage	Equation (6)	Equation (7)

**Table 2.** Parameters in the ray launching simulation.

**Frequency**		**2.41 GHz**
Vertical plane angle resolution	Δθ	0.5°
Horizontal plane angle resolution	Δφ	0.5°
Reflections		6
Transmitter Power		18 dBm
Cuboids resolution		0.5 m

**Table 3.** Parameters for the different considered wireless communication systems.

	**ZigBee Xbee Pro**	**ZigBee Xbee**	**Bluetooth-Low Energy**		**Bluetooth Class 1**	**Bluetooth Class 2**	**Bluetooth Class 3**
Transmitted power	10 dBm	10 dBm	−20 dBm	10 dBm	20 dBm	4 dBm	0 dBm
Sensitivity	−100 dBm	−92 dBm	−93 dBm	−87 dBm	−90 dBm	−90 dBm	−90 dBm

**Table 4.** Configuration of the parameters of the XBee Pro wireless devices to measure the received power level.

**Transmitted Power**	**18 dBm (Maximum Default Value)**
Transmission rate	57,600 bps
Frequency	2.41 (ZigBee Channel 12)
Measurement time	5 min

**Table 5.** Configuration of the parameters of the XBee Pro wireless devices to measure the ZigBee radio link quality.

**Transmitted Power**	**10 dBm (Maximum Default Value)**
Transmission rate	57,600 bps
Frequency	2.41 (ZigBee Channel 12)
Transmitted packet	100,000
